# Factors associated with elevated alanine aminotransferase in employees of a German chemical company: results of a large cross-sectional study

**DOI:** 10.1186/s12876-021-01601-2

**Published:** 2021-01-09

**Authors:** Matthias Claus, Christoph Antoni, Bernd Hofmann

**Affiliations:** 1 Corporate Health Management, ESG/CS - H308, BASF SE, 67056 Ludwigshafen am Rhein, Germany; 2 Department of Medicine II, University Medical Center Mannheim, Medical Faculty Mannheim, Heidelberg University, 68167 Mannheim, Germany

**Keywords:** Alanine aminotransferase, Germany, Non-alcoholic fatty liver disease, Quantile regression, Serum transaminase, Cross-sectional study, Liver enzymes, Social determinants, Prevalence, Employees

## Abstract

**Background:**

We aimed to determine the prevalence of elevated alanine aminotransferase (eALT) in employees of a German chemical company, and analyze its association with sociodemographic, work- and lifestyle-related factors.

**Methods:**

The cross-sectional study is based on data surveyed from occupational health check-ups between 2013 and 2018 at the site clinic of a chemical company based in Ludwigshafen, Germany. We used logistic regression analyses to assess the association between sociodemographic, work- and lifestyle-related characteristics and eALT. Quantile regression technique was applied to investigate if associations vary across different quantiles of the ALT distribution.

**Results:**

Participants (n = 15,348) were predominantly male (78.3%) with a mean age of 42.2 years (SD 10.7). The prevalence of eALT was 18.5% (21.6% in men/7.2% in women) with a geometric mean of 28.9 U/L (32.8 U/L in men/18.5 U/L in women). In the multivariable logistic regression model, odds of eALT were significantly higher for males (OR 2.61; 95%-CI 2.24–3.05), manual workers (OR 1.23; 95%-CI 1.06–1.43), overweight (OR 2.66; 95%-CI 2.36–3.00) or obese respondents (e.g. OR 7.88; 95%-CI 5.75–10.80 for obesity class III), employees who consume any number of alcoholic drinks/week (e.g. OR 1.32; 95%-CI 1.16–1.49 for ≥ 3 drinks per week) and diabetics (OR 1.47; 95%-CI 1.22–1.78). Additionally, season of participation was significantly associated with eALT, with odds being higher for participation in spring, fall or winter, as compared to summer. A significant interaction between age and gender (*p*_Interaction_ < 0.001) was found, showing approximately a u-shaped age/ALT relationship in women and an inversely u-shaped relationship in men. Quantile regression showed an increasing positive effect of male gender, overweight/obesity, and for diabetics on ALT level when moving from the lowest (q0.1) to the highest (q0.9) considered quantile. Additionally, from the lowest to the highest quantile an increasing negative effect on ALT for older age was observed.

**Conclusions:**

Prevalence of eALT in our sample of employees can be considered as high, with almost one in five participants affected. Identification of risk groups allows the implementation of targeted preventive measures in order to avoid transition to severe morbidity.

## Background

Alanine aminotransferase is a transaminase enzyme present in serum and organ tissue (especially the liver), and a commonly used marker for hepatocellular injury. In the absence of excessive alcohol consumption and viral hepatitis, elevated alanine aminotransferase (eALT) is considered an indicator for non-alcoholic fatty liver disease (NAFLD) which is defined as hepatic steatosis with a fat content of more than 5% of the liver weight or macrosteatosis of hepatocytes of the same degree in the absence of secondary causes of hepatic fat accumulation. NAFLD encompasses a variety of pathological conditions ranging from asymptomatic steatosis to non-alcoholic steatohepatitis (NASH), fibrosis and the potentially fatal liver cirrhosis.

NAFLD is the most common liver disease in Western countries, with approximately one in four persons affected in Europe and the USA according to a recent systematic review and meta-analysis [1]. Thus, NAFLD poses a major public health challenge and points to an enormous potential for prevention. Generally, liver biopsy is considered the gold standard for the diagnosis of NAFLD. Due to its invasiveness and cost however, it is not a feasible method in screening for NAFLD in large asymptomatic populations. Imaging techniques as ultrasonography are equally costly and time-consuming. Instead, available guidelines recommend the use of eALT as an economical and non-invasive alternative for the diagnosis of NAFLD in large-scale epidemiological studies whenever imaging tools are not feasible [2]. Components of metabolic syndrome (e.g. obesity, insulin resistance, dyslipidemia) have been consistently described as being associated with eALT/NAFLD [3, 4]. Evidence suggests a reciprocal relationship between these entities, with features of metabolic syndrome being more frequent in persons with eALT/NAFLD but also increasing the risk for developing eALT/NAFLD [5, 6]. Furthermore, sociodemographic characteristics (e.g. age [7–12], gender [9, 11–16], ethnicity [9, 10, 14, 17]), occupational factors (e.g. shift work [18–21]), and genetic predisposition [22, 23] have been linked to eALT/NAFLD in recent studies, although their exact role remains to be clarified.

To the best of our knowledge, comprehensive data on the prevalence of eALT is currently unavailable for Germany. With the current study, our intention is to add to the ever-growing body of literature using a large dataset from occupational health check-ups in a German company. We aimed to determine the prevalence of eALT in a sample of employees and analyze its association with sociodemographic, work- and lifestyle-related factors. Additionally, using quantile regression technique, our aim was to examine if associations are stable across different quantiles of the ALT distribution.

## Methods

### Study design

The cross-sectional study is based on data collected during occupational health check-ups between January 2013 and December 2018 at the site clinic of a chemical company based in Ludwigshafen am Rhein, Germany. At the Ludwigshafen site of the company, approximately 35,000 employees are currently employed on an area of about 10 km^2^, with approximately 200 industrial plants and about 2000 buildings. The occupational health check-up was introduced in the year 2011 at the Ludwigshafen site of the company and aims at the early detection of chronic diseases. It serves as a supplement to periodic, mandatory occupational health surveillance examinations. Participation in the check-up is entirely voluntary and can be repeated every three years. All employees are personally invited to the check-up by email. Completion of a written questionnaire, a venous blood draw and a comprehensive physical examination including anamnesis and documentation of health behavior by an occupational health physician are the main components of the check-up. Due to a change of laboratory equipment (clinical chemistry analyzer) in December 2012, data from check-ups surveyed between 2011 and 2012 were excluded. If an employee received multiple check-ups during the period of subject recruitment, only first-time visits were considered. Trainees, persons with missing values regarding ALT, pregnant women, participants with a known chronic liver disease other than NAFLD, and employees with known alcohol abuse were excluded from all analyses. Specifically, we excluded participants with alcoholic liver disease (ICD-10: K70; F10), significant alcohol consumption exceeding 21 or 14 standard drinks per week in respectively men and women as defined by the AASLD guideline on the diagnosis and management of NAFLD [24], those with drug-induced/toxic liver disease (ICD-10: K71), metabolic liver disease (ICD-10: E83.0; E83.1), autoimmune liver disease (ICD-10: K74.3; K75.4; K83.0), infectious liver disease/viral hepatitis (ICD:10: B15-B19; K77.0) or inflammatory liver disease (ICD-10: K75.0-K75.3). The ethical committee of the medical association of the German State of Rhineland-Palatinate has approved the retrospective analysis of routine data generated during the check-ups.

### Alanine aminotransferase (ALT)

A non-fasting venous blood sample was drawn from participants on the day of participation, which was centrifuged and analyzed within 24 h at a central hospital laboratory. Serum ALT was measured using an Architect ci8200 analyzer (Abbott Laboratories, Abbott Park, IL, USA). During subject recruitment (2013–2018), all serum samples were analyzed in the same laboratory with the same reference values. The limit of detection (LOD) for ALT was 6 U/L. The laboratory-specific upper limit of normal ALT activity was 45 U/L for males and 34 U/L for females.

### Covariates

Sociodemographic (age, gender) and occupational-related variables (occupational group, working time system) were directly extracted from employee records. Participants’ age at the time of examination was categorized into 5-year intervals (< 35, 35–39, …, ≥ 55). Occupational group (manual workers, skilled/supervisory workers, managerial staff) roughly represents the socioeconomic status of employees, with manual workers working in production lines and representing physical labor jobs, skilled/supervisory workers having a comparatively better education and performing commercial activities or office work, and managerial staff working in leading positions and usually holding an academic degree. Concerning working time system, we distinguish between day and shift workers. Most shift workers (> 90% in 2013 at the Ludwigshafen site) work 4 × 12 h-rotating shift schedules, with shifts lasting from 6 am to 6 pm followed by a leisure time of 24 h and the following shift beginning at 6 pm and ending at 6am with a subsequent leisure time of 48 h. During medical anamnesis, the responsible occupational health physician ascertained smoking status (smoker, former smoker, non-smoker), alcohol consumption (drinks/week), as well as height and weight of all participants. We calculated body mass index (BMI; *weight [in *kg*]/height squared [in *m^2^*]*) and categorized participants according to the classification provided by the World Health Organization (normal weight: BMI < 25 kg/m^2^, overweight: 25 kg/m^2^ ≤ BMI < 30 kg/m^2^, obesity class I: 30 kg/m^2^ ≤ BMI < 35 kg/m^2^, obesity class II: 35 kg/m^2^ ≤ BMI < 40 kg/m^2^, obesity class III: BMI ≥ 40 kg/m^2^). Alcohol consumption was surveyed by self-reported number of drinks per week (without specification of the exact amount or type of drink) and categorized into < 1 drink/week, 1 drink/week, 2 drinks/week, and 3 or more drinks/week. History of diabetes mellitus (DM) was considered present if any of the following was true: HbA_1c_ ≥ 6.5%, diagnosis made by a physician, self-reported diabetes in the check-up questionnaire. In addition, we considered weekday (Monday, Tuesday, …, Weekend), season (Spring [March–May], Summer [June–August], Fall [September–November], Winter [December–February]), and year of examination (2013–2018) in our analyses.

### Statistical analyses

Absolute and relative frequencies were used for a general description of the sample. Owing to the skewed distribution of the outcome, we used the median (with interquartile range [IQR]) and the geometric mean (with geometric standard deviation factor [GSD]) as measures of central tendency and dispersion. To calculate the geometric mean and GSD, ALT concentrations below the LOD (< 6 U/L; n = 14 [0.1%]) were substituted by the LOD divided by the square root of 2, a commonly applied method of imputation [25]. Univariable and multivariable logistic regression models were estimated to analyze the association between sociodemographic, lifestyle-, and work-related factors with elevated ALT. All variables being significantly associated with ALT in univariable analyses (*p* < 0.05) were considered in the final multivariable logistic regression model. We provided unadjusted and adjusted odds ratios ([a]OR) as measures of effect and corresponding 95%-confidence intervals (95%-CI). Due to reported gender disparities in existing studies on the association of considered factors and elevated ALT [11, 15, 26], we tested for significant multiplicative interactions between sex and all other covariates included in the final model using likelihood ratio tests. A significant interaction between categorical age and sex (*p*_Interaction_ < 0.001) was found. We used STATA’s *margins* and *marginsplot* commands to decipher and visualize the interaction. In addition, quantile regression technique [27] was applied to estimate the association of the same covariates included in the final multivariable logistic regression model (with and without the age/gender interaction) at selected quantiles (0.1, 0.25, 0.5 [median], 0.75, 0.9) of the outcome using STATA’s *sqreg* command with bootstrapped standard errors (500 repetitions). Quantile regression can be used to model effects of covariates across selected quantiles (including the median) of the outcome variable (here: ALT) in order to gain a deeper insight of the associations especially at the tails of the distribution. Compared to conventional linear regression models, quantile regression is more robust to outliers and does not require assumptions on the distribution of residuals or the outcome. Thus, transformations of the skewed outcome variable are not necessary and, unlike analyses on log-transformed outcomes, its estimates are directly interpretable. In this regard, estimated coefficients at a certain quantile quantify the change in the outcome variable per one-unit change in the covariate. We used Wald-tests to formally test for equality of estimates across the five considered quantiles. Results of the quantile regression are graphically displayed using the user-written *grqreg* [28] STATA-command. Complete case analysis was applied to deal with missing values in covariates (n = 616; 4.0%) when performing the regression analyses. A *p*-value < 0.05 was considered statistically significant. Due to the exploratory character of the present analysis, we did not adjust for multiple testing. STATA/SE 15.1 (StataCorp LLC, College Station, TX, USA) was used for all statistical analyses.

## Results

Altogether, 15,939 employees were first-time participants in the occupational health check-up between 2013 and 2018. Trainees (n = 208), persons with missing values in the outcome (n = 81), pregnant women (n = 61), participants with alcoholic liver disease (ICD-10: K70; F10; n = 146) or significant alcohol consumption (n = 6), those with drug-induced/toxic liver disease (ICD-10: K71; n = 7), metabolic liver disease (ICD-10: E83.0; E83.1; n = 18), autoimmune liver disease (ICD-10: K74.3; K75.4; K83.0; n = 2), infectious liver disease/viral hepatitis (ICD:10: B15–B19; K77.0; n = 61) or inflammatory liver disease (ICD-10: K75.0–K75.3; n = 1) were excluded leading to a final sample of 15,348 persons. Participants were predominantly male (78.3%) with a mean age of 42.2 years (SD 10.7; range 18–65). Most employees had regular daytime working hours (71.2%) and were employed as manual (35.5%) or skilled/supervisory workers (42.0%). Regarding lifestyle-related factors one in five respondents were smokers (19.7%) and/or obese (19.1%). More than a quarter of participants (28.0%) stated to consume less than one alcoholic drink per week (Table 1).

**Table 1:** Prevalence of elevated ALT stratified by sociodemographic, work- and lifestyle-related factors (n = 15,348)

	n (%)	% ALT elevated men: > 45 U/L women: > 34 U/L	Geometric mean U/L (GSD)	Median U/L (IQR)
Total	15,348 (100.0)	18.5	28.9 (1.65)	28 (21–40)
Sociodemographic factors				
Age (in years)				
< 35	4030 (26.3)	17.2	27.1 (1.70)	26 (19–38)
35–39	1818 (11.9)	19.8	28.9 (1.70)	28 (20–40)
40–44	2200 (14.3)	18.5	28.2 (1.68)	28 (20–40)
45–49	2742 (17.9)	19.5	30.1 (1.63)	29 (22–41)
50–54	2534 (16.5)	18.9	30.6 (1.56)	30 (23–40)
≥ 55	2024 (13.2)	18.2	30.2 (1.57)	29 (22–39)
Gender				
Male	12,013 (78.3)	21.6	32.8 (1.56)	31 (24–43)
Female	3335 (21.7)	7.2	18.5 (1.52)	18 (14–23)
Work-related factors				
Working time system				
Day work	10,931 (71.2)	16.4	27.5 (1.66)	27 (19–38)
Shift work	4417 (28.8)	23.6	32.9 (1.59)	32 (24–44)
Occupational group				
Manual worker	5447 (35.5)	24.0	32.8 (1.61)	32 (24–44)
Skilled/supervisory worker	6453 (42.0)	16.8	27.3 (1.67)	27 (19–38)
Managerial staff	3448 (22.5)	13.1	26.5 (1.61)	26 (19–35)
Lifestyle-related factors				
Smoking status^a^				
Non-smoker	8735 (56.9)	16.6	27.6 (1.65)	27 (20–38)
Former smoker	3552 (23.1)	23.3	32.2 (1.62)	32 (23–44)
Smoker	3023 (19.7)	18.6	29.5 (1.63)	29 (21–40)
Body-mass-index (kg/m^2^)^a^				
Normal weight (< 25)	6138 (40.0)	7.8	23.1 (1.57)	23 (17–30)
Overweight (25– < 30)	6281 (40.9)	20.1	31.5 (1.57)	31 (23–42)
Obesity class I (30– < 35)	2150 (14.0)	36.4	38.0 (1.62)	38 (28–53)
Obesity class II (35– < 40)	570 (3.7)	40.7	39.9 (1.71)	39.5 (28–56)
Obesity class III (≥ 40)	207 (1.4)	40.1	40.5 (1.65)	37 (30–56)
Alcohol consumption^a^ (drinks per week)				
< 1	4297 (28.0)	16.5	27.1 (1.67)	26 (19–37)
1	3911 (25.5)	18.4	28.2 (1.66)	27 (20–39)
2	3294 (21.5)	18.3	29.7 (1.61)	29 (22–40)
≥ 3	3256 (21.2)	21.7	31.9 (1.61)	31 (23–43)
Diabetes				
History of diabetes mellitus				
No	14,711 (95.9)	17.8	28.6 (1.64)	28 (21–39)
Yes	637 (4.2)	35.5	37.4 (1.68)	36 (27–53)
Time of examination				
Day of week				
Monday	2920 (19.0)	18.5	28.6 (1.65)	28 (21–40)
Tuesday	3717 (24.2)	18.5	28.7 (1.65)	28 (20–40)
Wednesday	3170 (20.7)	18.6	29.1 (1.66)	28 (21–40)
Thursday	3074 (20.0)	18.9	29.5 (1.62)	29 (21–39)
Friday	2465 (16.1)	18.0	28.8 (1.66)	28 (20–39)
Weekend	2 (0.0)	–	–	–
Season				
Spring (March–May)	4175 (27.2)	20.2	29.9 (1.65)	29 (21–41)
Summer (June–August)	3589 (23.4)	15.1	27.2 (1.63)	27 (20–37)
Fall (September–November)	3291 (21.4)	17.2	28.4 (1.64)	28 (20–39)
Winter (December–February)	4293 (28.0)	20.8	30.0 (1.66)	29 (21–41)
Year				
2013	5778 (37.7)	17.6	28.4 (1.65)	28 (20–39)
2014	2471 (16.1)	20.2	30.3 (1.63)	30 (22–41)
2015	1232 (8.0)	20.6	29.7 (1.61)	29 (21–40)
2016	1628 (10.6)	20.4	29.6 (1.68)	29 (21–40)
2017	1897 (12.4)	15.5	27.8 (1.63)	27 (20–38)
2018	2342 (15.3)	19.0	29.0 (1.67)	28 (21–39)

GSD, geometric standard deviation factor, dimensionless, to be multiplied/divided by the geometric mean; IQR, interquartile range

^a^Missing values for smoking status (n = 38), BMI (n = 2) and alcohol consumption (n = 590) have been omitted in the table

The average ALT level in our sample was 28.9 U/L and 28 U/L (for the geometric mean and median respectively; range < LOD-621 U/L), with an overall prevalence of elevated ALT of 18.5%. The prevalence was considerably higher in men (21.6%) than women (7.2%). Additionally, a comparatively high prevalence was found in manual and shift workers (24.0% and 23.6% respectively), former smokers (23.3%), respondents consuming three or more alcoholic drinks per week (21.7%), obese participants (36.4% in obesity class I, 40.7% in class II, 40.1% in class III), and respondents with a history of DM (35.5%; Table 1). Apart from weekday of participation, all covariates were significantly associated with eALT in univariable logistic regression analyses and thus considered in the final multivariable model (Table 2).

**Table 2:** Logistic regression analyses regarding the association of sociodemographic, work- and lifestyle-related factors with eALT (n = 14,732)

	Logistic regression (n = 14,732)	
	Univariable	Multivariable
	OR (95%-CI)	aOR (95%-CI)
Sociodemographic factors		
Age (in years)		
< 35	Reference	Reference
35–39	1.21 (1.05–1.40)	1.02 (0.88–1.20)
40–44	1.12 (0.97–1.28)	0.88 (0.76–1.03)
45–49	1.21 (1.06–1.37)	0.82 (0.71–0.94)
50–54	1.14 (1.00–1.30)	0.67 (0.58–0.77)
≥ 55	1.08 (0.93–1.24)	0.58 (0.50–0.69)
Gender		
Male	3.50 (3.04–4.03)	2.61 (2.24–3.05)
Female	Reference	Reference
Work-related factors		
Working time system		
Day work	Reference	Reference
Shift work	1.55 (1.42–1.69)	0.93 (0.84–1.04)
Occupational group		
Manual worker	2.02 (1.80–2.28)	1.23 (1.06–1.43)
Skilled/supervisory worker	1.31 (1.16–1.47)	1.07 (0.94–1.22)
Managerial staff	Reference	Reference
Lifestyle-related factors		
Smoking status		
Non-smoker	Reference	Reference
Former smoker	1.52 (1.38–1.68)	1.05 (0.94–1.18)
Smoker	1.13 (1.01–1.26)	0.83 (0.74–0.94)
Body-mass-index (kg/m^2^)		
Normal weight (< 25)	Reference	Reference
Overweight (25– < 30)	3.05 (2.72–3.42)	2.66 (2.36–3.00)
Obesity class I (30– < 35)	6.79 (5.95–7.74)	6.10 (5.29–7.02)
Obesity class II (35– < 40)	8.45 (6.95–10.28)	7.75 (6.30–9.54)
Obesity class III (≥ 40)	8.19 (6.07–11.06)	7.88 (5.75–10.80)
Alcohol consumption (drinks per week)		
< 1	Reference	Reference
1	1.15 (1.02–1.28)	1.21 (1.07–1.37)
2	1.13 (1.01–1.28)	1.14 (1.00–1.30)
≥ 3	1.41 (1.25–1.58)	1.32 (1.16–1.49)
Diabetes		
History of diabetes mellitus		
No	Reference	Reference
Yes	2.45 (2.06–2.91)	1.47 (1.22–1.78)
Time of examination		
Day of week^a^		
Monday	Reference	^b^
Tuesday	0.98 (0.87–1.12)	^b^
Wednesday	1.00 (0.87–1.14)	^b^
Thursday	1.02 (0.90–1.17)	^b^
Friday	0.98 (0.85–1.13)	^b^
Weekend	–	^b^
Season		
Spring (March–May)	1.43 (1.27–1.62)	1.40 (1.23–1.59)
Summer (June–August)	Reference	Reference
Fall (September–November)	1.17 (1.02–1.33)	1.21 (1.06–1.40)
Winter (December–February)	1.47 (1.31–1.66)	1.49 (1.31–1.69)
Year		
2013	Reference	Reference
2014	1.18 (1.05–1.33)	1.00 (0.88–1.14)
2015	1.20 (1.02–1.40)	1.09 (0.92–1.28)
2016	1.22 (1.06–1.40)	1.19 (1.02–1.38)
2017	0.87 (0.76–1.01)	0.84 (0.72–0.98)
2018	1.08 (0.95–1.23)	1.05 (0.91–1.20)

(a)OR, (adjusted) odds ratio; 95%-CI, 95%-confidence interval

^a^Category "weekend" contained only two observations and was discarded when performing regression analyses

^b^Variable not considered in multivariable logistic regression model

**Fig. 1:**
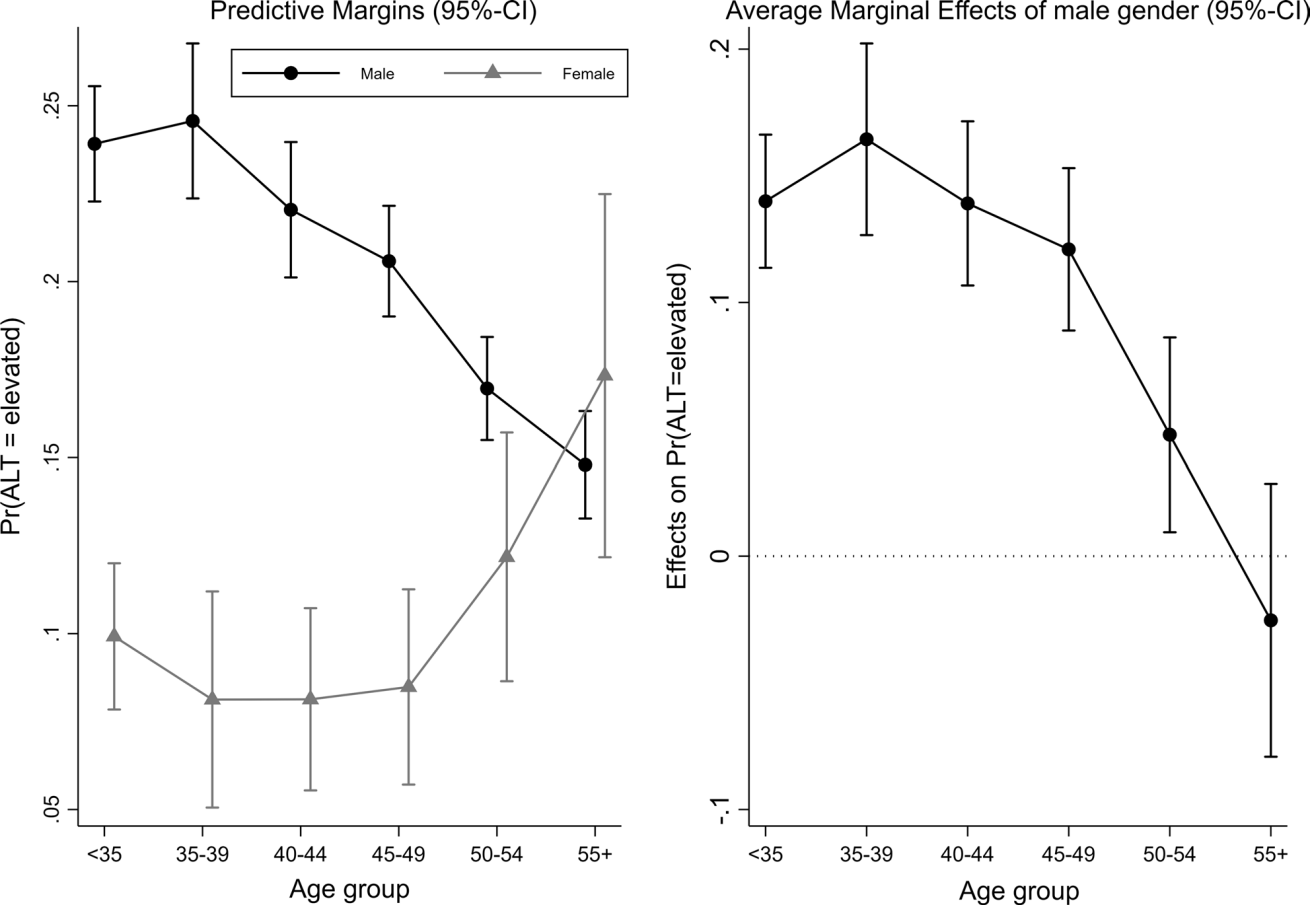
Interaction of categorical age and gender on eALT from multivariable logistic regression analysis (n = 14,732)

In the multivariable model, the odds of eALT were significantly higher for males (vs. females), manual workers (vs. managerial staff), overweight employees or those with any class of obesity (vs. normal weight), respondents who consumed ≥ 1 alcoholic drink per week (vs. < 1 drink), participants with a history of DM, if participation took place in spring, fall, or winter (vs. summer), and participation in 2016 (vs. 2013). In contrast, odds of eALT were significantly lower for smokers (vs. non-smokers) and participation in 2017 (vs. 2013). Regarding age, compared to the youngest considered age group (< 35 years), odds of eALT increased by a factor of 1.02 (95%-CI 0.88–1.20) in respondents aged 35–39 years, and continuously decreased thereafter by a factor of 0.88 (95%-CI 0.76–1.03) in persons aged 40–44 years, 0.82 (95%-CI 0.71–0.94) in those aged 45–49 years, 0.67 (95%-CI 0.58–0.77) in those aged 50–54 years, and 0.58 (95%-CI 0.50–0.69) in the oldest age group (≥ 55 years). We found a significant interaction effect between gender and categorical age (*p*_Interaction_ < 0.001) visualized in Fig. 1. The adjusted predicted probability of eALT in males slightly increased from the youngest (< 35 years) to the 35–39 years age group and continuously decreased thereafter. In women there was a decrease from the youngest (< 35 years) to the 35–39 years age group and a sharp increase at age 50–54 (left-hand side of Fig. 1).

**Fig. 2:**
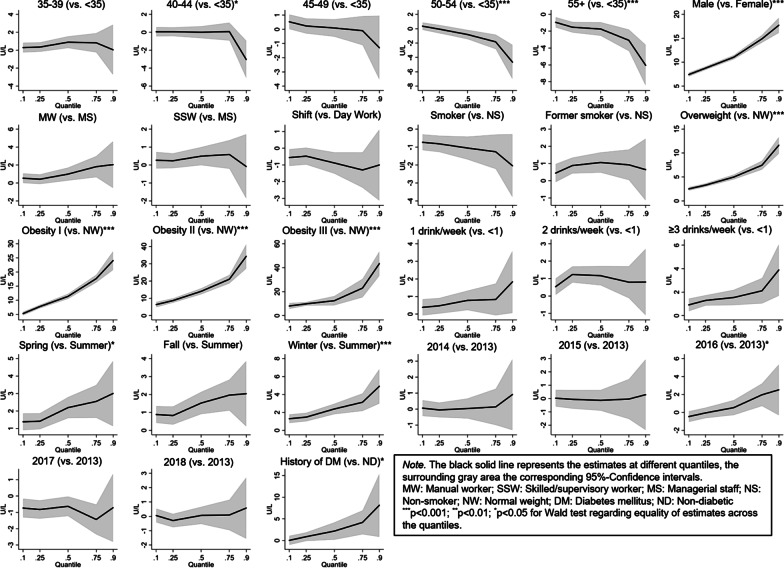
Results of the quantile regression model showing coefficients at different quantiles of the ALT-outcome (n = 14,732)

Correspondingly, the average marginal effect of male gender on the probability of an elevated ALT level (i.e. the difference in adjusted predicted probabilities between males and females) increased from < 35 years to 35–39 years and continuously decreased thereafter (right-hand side of Fig. 1). Compared to the corresponding reference groups, results from the multivariable quantile regression model (Fig. 2; Additional file 1) showed significantly higher values at the median (q0.50) of the outcome distribution for males (11.05 U/L; 95%-CI 10.58–11.51), manual workers (0.98 U/L; 95%-CI 0.24–1.73), former smokers (1.06 U/L; 95%-CI 0.46–1.66), overweight (4.98 U/L; 95%-CI 4.45–5.51) and obese participants (11.38 U/L; 95%-CI 10.43–12.33 for obesity class I; 14.06 U/L; 95%-CI 12.24–15.87 for class II; 12.47 U/L; 95%-CI 8.46–16.48 for class III), consumers of one or more alcoholic drinks per week (0.78 U/L; 95%-CI 0.22–1.34 for 1 drink/week; 1.17 U/L; 95%-CI 0.60–1.73 for 2 drinks/week; 1.54 U/L; 95%-CI 0.88–2.21 for ≥ 3 drinks/week), if participation took place in spring (2.19 U/L; 95%-CI 1.58–2.80), fall (1.52 U/L; 95%-CI 0.89–2.15) or winter (2.38 U/L; 95%-CI 1.78–2.98), and for participants with a history of DM (2.18 U/L; 95%-CI 0.15–4.22). Contrarily, significantly lower ALT values were observed for shift workers (− 0.88 U/L; 95%-CI − 1.53; − 0.23), smokers (− 1.06 U/L; 95%-CI − 1.70; − 0.41), and participation in 2017 (− 0.63 U/L; 95%-CI − 1.24; − 0.02). With regard to age, compared to participants younger than 35 years, significantly higher values were observed for age group 35–39 years (0.90 U/L; 95%-CI 0.23–1.57), and lower values for age groups 50–54 years (− 0.81; 95%-CI − 1.47; − 0.14) and ≥ 55 years (− 1.74; 95%-CI − 2.56; − 0.91). Regarding heterogeneity in the association between covariates and ALT levels across different quantiles of the outcome distribution, we observed a sharp monotonic increase in the positive effect of male gender on ALT level, when moving from the lowest (q0.10) to the highest (q0.90) considered ALT quantile (*p* < 0.001 for Wald-test regarding equality of estimates across quantiles). For instance, ALT values of males were 7.41 U/L (95%-CI 6.98–7.85) higher compared to females at the lower end of the ALT distribution (at q0.10) and 17.73 U/L (95%-CI 16.11–19.35) at the upper end (at q0.90). In a similar manner, from lowest to highest ALT quantile there was a monotonous and strong increase in the positive effect of being overweight or obese (*p* < 0.001 for all BMI categories) and in employees with a history of DM (*p* < 0.05). For participants with a BMI ≥ 40 kg/m^2^ (obesity class III) for example, ALT values were 8.03 U/L (95%-CI 5.43–10.62) higher compared to normal weight individuals at the lowest considered quantile of the ALT distribution (q0.10) and 43.54 U/L (95%-CI 33.14–53.93) at the highest (q0.90). In contrast, an increasing negative effect was visible for age categories 50–54 years and ≥ 55 years (vs. < 35 years; *p* < 0.001).

**Fig. 3:**
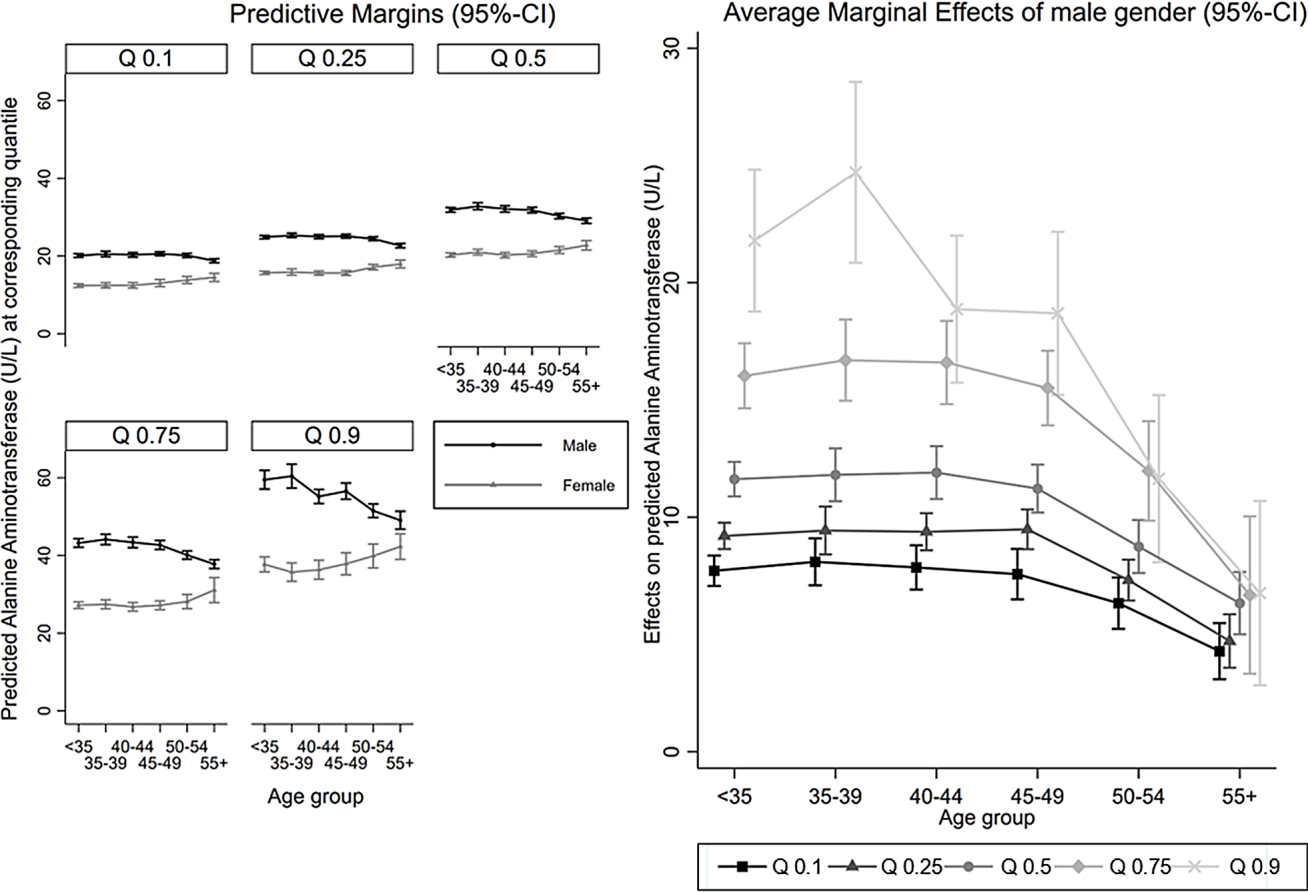
Interaction of categorical age and gender in the quantile regression model (n = 14,732)

Re-estimation of the same quantile regression model including the interaction between categorical age and gender showed approximately an inverse u-shaped relationship between age and adjusted predicted ALT levels in males, becoming more pronounced when moving from the lowest to the highest considered quantile. In females, there was an increase in the predicted ALT level from 45 to 49 years upwards for all quantiles, and a u-shaped relationship at the highest quantile (left-hand side of Fig. 3).

Accordingly, there was a positive effect of male gender on ALT across all considered age categories and quantiles (right-hand side of Fig. 3). When moving from the lowest to the highest age group however, this effect declined from the 45–49 years age group and onwards for all quantiles. The strongest decrease was found at the highest quantiles such that in respondents aged 55 years or older, the difference in the effect of male gender on ALT across quantiles was comparatively small.

## Discussion

### Main findings

We aimed to investigate the prevalence of eALT in employees of a chemical company, and analyze its association with sociodemographic, work- and lifestyle-related factors. The overall prevalence of eALT among the employees in the cohort was 18.5% (21.6% in males/7.2% in females). After multivariable adjustment, odds of eALT were comparatively higher for men, manual workers, overweight/obese participants, consumers of one or more alcoholic drinks per week, diabetics, and if participation took place in spring, fall or winter (vs. summer). Contrarily, odds of eALT were comparatively lower with increasing age and for smokers. There was a significant interaction between age and gender, yielding approximately an inverse u-shaped age/ALT relationship in men and a u-shaped relationship in women. Quantile regression showed that associations between considered factors and ALT level vary widely across quantiles. There was e.g. a sharp increase in the positive effect of male gender and overweight/obesity on ALT levels, when moving from the lowest to the highest considered quantile.

### Implications

Considering the prevalence of eALT in our sample of employees (18.5%) in the context of the existing body of literature has to be done with caution since studies may vary widely according to thresholds for elevated ALT, distribution of risk factors within the population studied and diagnosis of NAFLD (e.g. via imaging procedures, liver enzymes or biopsy). A recent systematic review and meta-analysis on the prevalence of NAFLD (diagnosed via imaging techniques) in adults found a global prevalence of 25.2% (45 studies/371,876 participants), and a corresponding estimate of 23.7% for Europe (11 studies/16,735 persons) [1]. With regards to Germany, results from the population-based Study of Health in Pomerania (SHIP) on adults aged 20–79 years reported an estimated ultrasonography diagnosed NAFLD prevalence of 29.9% (n = 4222) [29].

The finding of a higher prevalence of eALT in men compared to women aligns with earlier studies on the topic [9, 11–16]. Notably, there were profound differences in the association of age and ALT by sex. In males, the adjusted predicted probability of eALT peaks between 35–39 years and continuously declines thereafter. In women however, this probability remains at a comparatively low level with a sharp increase beginning at the age of 50. A recent review by Ballestri and colleagues summarized the evidence of earlier studies on sex-specific associations between age and eALT/NAFLD [30]. Similar to our findings, an inverted u-shaped curve was reported for men (peaking between 40 and 49 years) [30–32], and comparatively sharp increases in ALT after the age of 50 years in women [30–33]. It has been hypothesized that age-related declines in ALT might be caused by changes in volume or function of the aging liver [7, 15, 34]. The increase of ALT in women aged 50 years and older has been attributed to diminishing protective effects of estrogens on the liver after menopause (e.g. by inhibiting mitochondrial dysfunction and cellular senescence) [15, 30, 31, 35]. In a study by Park et al. for example, odds of NAFLD in postmenopausal women were 1.71-times (95%-CI 1.27–2.32; n = 2644) the odds of premenopausal women [36].

Regarding occupational factors, several studies examined the effect of shift work on liver enzymes [18–21]. In a cross-sectional study by Choi et al. (n = 21,951), a significant effect of shift work on eALT was found in females only. After stratification into subgroups of shift work pattern, no significant effect of any type of shift work (compared to day work) on ALT was found [18]. Wang et al. examined the relationship between night shift work and eALT in males (n = 4740) using a prospective study design. According to the authors, night shift workers had an increased risk of eALT compared to day workers [19]. In a further retrospective cohort study, Lin et al. examined the effect of long-term rotating shift work exposure on the normalization of plasma ALT levels over a 5-year period (n = 275). The authors reported that after multivariable adjustment, workers exposed to rotating shift work were less likely to normalize ALT levels within 5 years of follow-up [20]. In a further study on 758 employees by Lin et al. it was found that persistent rotating shift work exposure aggravates the development of eALT in workers with preexisting sonographic fatty liver [21]. In our study, we did not observe a significant effect of shift work on ALT. It should be noted however, that information on working time system was available for the time of participation only. Information on duration of shift work or changes from day to shift work or vice versa during time of employment was not available. Regarding occupational group we noted some type of gradient, with employees being more likely to present with eALT in lower occupational groups. This finding might be due to residual confounding by factors associated with ALT and occupational group that were not accounted for in our analysis (e.g. physical activity, nutritional factors, workplace environment/exposure to hepatotoxic chemicals). Regarding workplace environment, we believe, however, that the potential influence of exposure to hepatotoxic chemicals on ALT values in our sample is negligible since there are only few plants where these substances are handled, and existing safety measures are strict. Consequently, the number of manual workers with ALT values affected by hepatotoxic chemicals is presumably very low and we do not believe that this might explain the difference between occupational groups. Regarding BMI, our findings align with a variety of earlier studies on the topic where associations between overweight/obesity and eALT/NAFLD have been reported [11, 14–16, 37, 38]. Interestingly, we observed substantial heterogeneity in the effect of overweight/obesity across the entire ALT distribution, monotonically increasing when moving from the lowest to the highest considered quantile. Thus, employees within the highest quantiles of the ALT distribution might particularly benefit from weight reduction.

Compared to abstainers or persons consuming less than one alcoholic drink per week, any frequency of weekly alcohol consumption was significantly associated with ALT levels in multivariable regression models. Findings from the literature are conflicting, with several studies reporting an increase of liver enzymes with increasing alcohol consumption [13, 31, 38–40], whereas others did not observe any association [11, 41–43] or even found evidence for a hepatoprotective effect for light to moderate consumption of alcohol [44, 45].

The seemingly protective effect of smoking on ALT-levels found in both multivariable logistic and quantile regression models cannot be explained easily. Earlier studies reported conflicting results on this regard. Whereas some studies did not find effects of smoking on ALT/NAFLD [11, 39, 43, 46, 47], others claimed ALT was increased (in men and women [48] or women only [40]) or decreased [41].

The prevalence of eALT in participants with a history of diabetes mellitus (35.5%) was amongst the highest besides obese respondents. Significant associations between history of diabetes mellitus and ALT were found in multivariable regression analyses. The association of NAFLD/eALT and diabetes mellitus is well established in the available literature, although considerable debate remains as to which factor precedes the other. The relationship between these two disease entities has been described as bidirectional with each factor acting as a causative and also exacerbating factor for the other although the detailed underlying pathophysiological mechanism remains to be fully elucidated [5, 6, 49].

Surprisingly, we found considerable seasonal variation in the prevalence of eALT, ranging from 15.1% in summer to 20.8% in winter. These findings remained stable in the fully adjusted logistic and quantile regression models. In accordance to that, Miyake and colleagues found ALT values peaking in January and falling to a minimum in July in their time-series analyses on the seasonal variation of liver function tests in Japanese outpatients using a large dataset (> 1 mio. tests) [50]. The authors suggested that increased physical activity and/or higher temperatures in the summer season leads to reduced vascular tone which in turn causes haemodilution of intravascular substances [50]. Calculating median values of hematocrit levels by season within our sample, showed, however, only slight variability from 43.1% in summer to 43.8% in winter. Furthermore, climatic stress or hormonal changes in the winter season are suggested to be responsible for increased ALT levels [50].

For employees with eALT/NAFLD, a further work-up is recommended according to existing guidelines and special attention should be paid to those with a high risk of advanced fibrosis. Liver fibrosis is an important prognostic factor which is associated with liver-related morbidity and mortality [51]. As recommended in the major guidelines, non-invasive fibrosis scores as FIB-4 and NFS are useful diagnostic modalities in estimation of advanced fibrosis [2, 24]. They show acceptable diagnostic performance both in patients with elevated and normal transaminase levels [52]. Using FIB-4 for our sample, we found respectively 89.4%, 10.0% and 0.5% (0.1% missing) respondents with eALT to have low, indeterminate or high risk of advanced fibrosis using < 1.45 and > 3.25 as cut-off values.

### Limitations

There are limitations to our study which should be acknowledged. Firstly, the cross-sectional design of the current study impedes to draw any causal conclusions. In addition to that, information on lifestyle-related factors such as smoking and alcohol consumption are based solely upon the voluntary self-report of employees and was not objectively validated. It is e.g. possible that isolated cases of alcoholism were not disclosed by the employees and not documented during the medical anamnesis carried out by the responsible occupational physician. We believe this number however to be very low since we used a sample on an active workforce. Furthermore, alcohol consumption was surveyed as “drinks/week”, lacking an exact definition of type and amount of drinks consumed. Thus, inaccuracies in disclosure/misreporting of information cannot be ruled out. Additionally, eALT is an imperfect indicator of NAFLD, since a variety of other factors might be responsible for increased ALT activity and up to 25% of patients with confirmed NAFLD are found to have normal ALT levels according to a recent systematic review and meta-analysis [53]. Furthermore, ALT was measured only once although ALT levels may vary over time. A further point relates to the fact that we used the laboratory-specific thresholds of 45 U/L for males and 34 U/L for females as upper limit of normal ALT activity. The optimal choice of thresholds for the definition of elevated ALT levels is however far from clear and there is no generally accepted upper limit of normal. Depending on the characteristics of the population studied, different cut-offs have been recommended in the literature [54–56]. For this reason, we performed additional analyses using ALT as a continuous dependent variable via quantile regression models as opposed to a simple elevated vs. non-elevated variable in logistic regression. The results of the logistic and quantile regression models were however quite consistent. Additionally, some misclassification might have occurred with regards to history of diabetes, since we did not distinguish between type 1 and type 2 diabetes in our questionnaire. Finally, there might be other factors potentially associated with ALT levels (e.g. physical activity, time the blood sample was drawn, hepatotoxic medications/chemicals at the workplace, extrahepatic diseases, coffee consumption, substance abuse etc.) which were not surveyed during the check-up and have not been considered in the analyses.

## Conclusions

In summary, prevalence of eALT in our sample of employees can be considered as high, with almost one in five participants affected. We found elevated ALT levels to be associated with male gender, overweight and obesity, alcohol consumption, history of diabetes mellitus, lower occupational group, and season of study participation. Identification of risk groups allows the implementation of targeted preventive measures in order to avoid transition to severe morbidity.

## Supplementary Information

**Additional file 1.** Results of the quantile regression model showing coefficients at different quantiles of the ALT-outcome (n = 14,732).

## Data Availability

The datasets generated or analyzed during the current study are not publicly available due to company-specific data protection laws but are available from the corresponding author on reasonable request.

## Abbreviations

95%-CI: 95%-Confidence interval
AASLD: American Association for the Study of Liver Diseases
(a)OR: (Adjusted) odds ratio
BMI: Body-mass-index
DM: Diabetes mellitus
(e)ALT: (Elevated) alanine aminotransferase
FIB-4: Fibrosis-4 Index
GSD: Geometric standard deviation factor
IQR: Interquartile range
LOD: Limit of detection
NAFLD: Non-alcoholic fatty liver disease
NASH: Non-alcoholic steatohepatitis
NFS: NAFLD Fibrosis Score
SD: Standard deviation
