# Supplementary material for: Factors associated with elevated alanine aminotransferase in employees of a German chemical company: results of a large cross-sectional study
Source: BMC Gastroenterol. 2021 Jan 9;21:25. doi: 10.1186/s12876-021-01601-2 (PMC7797104; doi:10.1186/s12876-021-01601-2)
Supplement: Supplementary file 1 — Additional file 1. Results of the quantile regression model showing coefficients at different quantiles of the ALT-outcome (n = 14,732). [file 12876_2021_1601_MOESM1_ESM.docx]

**Additional file 1.** Results of the quantile regression model showing coefficients at different quantiles of the ALT-outcome (n=14,732)

|  | **Multivariable quantile regression (n=14,732)** | | | | | |
| --- | --- | --- | --- | --- | --- | --- |
|  | **Q 0.1** | **Q 0.25** | **Q 0.5 (Median)** | **Q 0.75** | **Q 0.9** | **p-value** |
| *Sociodemographic factors* | *aCoef.  (95%-CI)* | *aCoef.  (95%-CI)* | *aCoef.  (95%-CI)* | *aCoef.  (95%-CI)* | *aCoef.  (95%-CI)* |  |
| **Age (in years)** |  |  |  |  |  |  |
| <35 | Reference | Reference | Reference | Reference | Reference |  |
| 35-39 | 0.29 (-0.27;0.86) | 0.35 (-0.19;0.90) | 0.90 (0.23;1.57) | 0.83 (-0.26;1.92) | 0.05 (-2.78;2.88) | 0.42 |
| 40-44 | 0.06 (-0.48;0.59) | 0.06 (-0.45;0.57) | 0.01 (-0.67;0.69) | 0.08 (-0.94;1.09) | -3.04 (-5.16;-0.92) | 0.02 |
| 45-49 | 0.53 (0.01;1.05) | 0.24 (-0.31;0.78) | 0.09 (-0.53;0.72) | -0.09 (-1.12;0.94) | -1.30 (-3.58;0.97) | 0.52 |
| 50-54 | 0.35 (-0.19;0.89) | -0.06 (-0.66;0.54) | -0.81 (-1.47;-0.14) | -1.83 (-2.86;-0.80) | -4.67 (-7.10;-2.24) | <0.001 |
| ≥55 | -0.94 (-1.57;-0.31) | -1.53 (-2.18;-0.88) | -1.74 (-2.56;-0.91) | -3.08 (-4.37;-1.78) | -6.06 (-8.52;-3.60) | <0.001 |
| **Gender** |  |  |  |  |  |  |
| Male | 7.41 (6.98;7.85) | 8.76 (8.37;9.16) | 11.05 (10.58;11.51) | 14.89 (14.06;15.71) | 17.73 (16.11;19.35) | <0.001 |
| Female | Reference | Reference | Reference | Reference | Reference |  |
| *Work-related factors* |  |  |  |  |  |  |
| **Working time system** |  |  |  |  |  |  |
| Day work | Reference | Reference | Reference | Reference | Reference |  |
| Shift work | -0.56 (-1.07;-0.05) | -0.47 (-0.99;0.05) | -0.88 (-1.53;-0.23) | -1.30 (-2.37;-0.24) | -1.00 (-3.15;1.15) | 0.51 |
| **Occupational group** |  |  |  |  |  |  |
| Manual worker | 0.53 (-0.03;1.09) | 0.41 (-0.14;0.97) | 0.98 (0.24;1.73) | 1.81 (0.62;3.00) | 2.04 (-0.59;4.67) | 0.21 |
| Skilled/supervisory worker | 0.26 (-0.20;0.73) | 0.24 (-0.19;0.66) | 0.49 (-0.02;1.00) | 0.58 (-0.22;1.39) | -0.09 (-1.90;1.72) | 0.70 |
| Managerial staff | Reference | Reference | Reference | Reference | Reference |  |
| *Lifestyle-related factors* |  |  |  |  |  |  |
| **Smoking status** |  |  |  |  |  |  |
| Non-smoker | Reference | Reference | Reference | Reference | Reference |  |
| Former smoker | 0.44 (-0.10;0.98) | 0.88 (0.41;1.35) | 1.06 (0.46;1.66) | 0.92 (0.03;1.82) | 0.64 (-1.17;2.45) | 0.35 |
| Smoker | -0.74 (-1.18;-0.29) | -0.82 (-1.29;-0.36) | -1.06 (-1.70;-0.41) | -1.26 (-2.23;-0.30) | -2.05 (-3.82;-0.27) | 0.67 |
| **Body-mass-index (kg/m²)** |  |  |  |  |  |  |
| Normal weight (<25) | Reference | Reference | Reference | Reference | Reference |  |
| Overweight (25-<30) | 2.50 (2.11;2.89) | 3.29 (2.90;3.68) | 4.98 (4.45;5.51) | 7.45 (6.49;8.41) | 11.63 (9.84;13.42) | <0.001 |
| Obesity class I (30-<35) | 5.26 (4.56;5.97) | 7.76 (7.10;8.43) | 11.38 (10.43;12.33) | 17.66 (16.23;19.09) | 24.08 (20.68;27.48) | <0.001 |
| Obesity class II (35-<40) | 6.35 (4.73;7.97) | 8.71 (7.35;10.06) | 14.06 (12.24;15.87) | 21.08 (18.53;23.62) | 34.33 (27.12;41.55) | <0.001 |
| Obesity class III (≥40) | 8.03 (5.43;10.62) | 9.82 (8.19;11.46) | 12.47 (8.46;16.48) | 23.00 (15.13;30.87) | 43.54 (33.14;53.93) | <0.001 |
| **Alcohol consumption**  **(drinks per week)** |  |  |  |  |  |  |
| <1 | Reference | Reference | Reference | Reference | Reference |  |
| 1 | 0.38 (-0.09;0.85) | 0.47 (0.02;0.92) | 0.78 (0.22;1.34) | 0.83 (-0.08;1.74) | 1.84 (0.05;3.63) | 0.45 |
| 2 | 0.53 (0.05;1.01) | 1.24 (0.76;1.71) | 1.17 (0.60;1.73) | 0.79 (-0.14;1.72) | 0.80 (-1.13;2.73) | 0.06 |
| ≥3 | 0.91 (0.36;1.46) | 1.29 (0.84;1.75) | 1.54 (0.88;2.21) | 2.11 (1.02;3.21) | 3.90 (1.68;6.12) | 0.08 |
| *Diabetes* |  |  |  |  |  |  |
| **History of diabetes mellitus** |  |  |  |  |  |  |
| No | Reference | Reference | Reference | Reference | Reference |  |
| Yes | 0.03 (-1.05;1.11) | 0.88 (-0.15;1.91) | 2.18 (0.15;4.22) | 4.19 (1.39;6.99) | 8.18 (0.80;15.55) | 0.03 |
| *Time of Examination* |  |  |  |  |  |  |
| **Season** |  |  |  |  |  |  |
| Spring (March-May) | 1.38 (0.89;1.87) | 1.41 (0.95;1.88) | 2.19 (1.58;2.80) | 2.55 (1.60;3.49) | 3.00 (1.12;4.89) | 0.02 |
| Summer (June-August) | Reference | Reference | Reference | Reference | Reference |  |
| Fall (September-November) | 0.88 (0.41;1.36) | 0.82 (0.31;1.34) | 1.52 (0.89;2.15) | 1.96 (1.09;2.83) | 2.04 (0.20;3.88) | 0.06 |
| Winter (December-February) | 1.29 (0.80;1.79) | 1.47 (0.98;1.96) | 2.38 (1.78;2.98) | 3.13 (2.14;4.13) | 4.92 (2.97;6.87) | <0.001 |
| **Year** |  |  |  |  |  |  |
| 2013 | Reference | Reference | Reference | Reference | Reference |  |
| 2014 | 0.06 (-0.45;0.57) | -0.06 (-0.53;0.41) | 0.03 (-0.60;0.67) | 0.13 (-1.01;1.27) | 0.91 (-1.33;3.16) | 0.90 |
| 2015 | 0.03 (-0.61;0.66) | -0.06 (-0.77;0.65) | -0.13 (-0.92;0.66) | -0.04 (-1.54;1.47) | 0.29 (-2.38;2.97) | 0.99 |
| 2016 | -0.47 (-1.08;0.14) | -0.06 (-0.66;0.54) | 0.53 (-0.32;1.38) | 1.96 (0.69;3.23) | 2.51 (-0.35;5.38) | 0.01 |
| 2017 | -0.74 (-1.32;-0.15) | -0.82 (-1.39;-0.25) | -0.63 (-1.24;-0.02) | -1.43 (-2.36;-0.51) | -0.71 (-2.82;1.39) | 0.25 |
| 2018 | 0.06 (-0.43;0.55) | -0.29 (-0.77;0.18) | 0.06 (-0.54;0.67) | 0.09 (-0.98;1.17) | 0.58 (-1.57;2.73) | 0.47 |
| aCoef.: adjusted Coefficient (in U/L) at the respective quantile; 95%-CI: 95%-Confidence Interval; p-value for Wald-test regarding equality of coefficients across quantiles | | | | | | |
